# A preliminary investigation on the relationship between virtues and pathological internet use among Chinese adolescents

**DOI:** 10.1186/1753-2000-8-8

**Published:** 2014-03-04

**Authors:** Yonghong Zhang, Zhihan Yang, Wenjie Duan, Xiaoqing Tang, Fengchun Gan, Fei Wang, Jinxia Wang, Pengfei Guo, Ying Wang

**Affiliations:** 1 Center of Studies for Psychology and Social Development, Southwest University, Chongqing, China; 2 School of Cultural and Social Development Studies, Southwest University, Chongqing, China; 3 Department of Applied Social Studies, City University of Hong Kong, Hong Kong, China; 4 Faculty of Education, Southwest University, Chongqing, China; 5 Office of Academic Affairs, The Sixteenth Middle School of Changzhi City, Shanxi, China; 6 Chongqing Cultural Development Research Institute, Chongqing, China; 7 Party Committee Office, Hospital (T. C. M) Affiliated to Luzhou Medical College, Sichuan, China; 8 Institute of Psychology, Chinese Academy of Sciences, Beijing, China

**Keywords:** Virtue, Pathological Internet use, Vitality, Conscientiousness, Relationship

## Abstract

**Background:**

Pathological Internet Use (PIU) has become a global issue associated with the increasing number of Internet users. Previous studies concerned both the interpersonal and intrapersonal vulnerable factors and the corresponding models. However, a limited amount of research has explored the relationship between positive factors and PIU.

**Objective:**

The current investigation attempted to clarify the relationship between virtues and PIU among Chinese adolescents; it also sought to explore the specific contributions of the three virtues. Virtue was the core concept in positive psychology and the *Values in Action Classification*. A recent study demonstrated that there might be three universal virtues (relationship, vitality, and conscientiousness).

**Methods:**

A cross-sectional sample of adolescents aged 12-17 years were recruited in 2013. A total of 674 adolescents (males = 302, females = 372; junior high school = 296, senior high school = 378) from eight junior and senior high schools in four provinces of Mainland China completed a package of psychological inventories, including the Chinese Virtues Questionnaire (CVQ) and the Adolescent Pathological Internet Use Scale (APIUS). The mean age of the current sample was 15.10 years (*SD* = 1.81) with an average of 5.31 years’ length (*SD* = 2.09) of Internet use.

**Results:**

A total of 9.50% participants exhibited significant symptoms of PIU. Male students (*M*_male_ = 2.50) had significantly higher scores on PIU than female students (*M*_female_ = 2.25). Relationship (*β* = -.24) and conscientiousness (*β* = -.21) negatively predicted PIU, whereas vitality (*β* = .25) positively predicted PIU. Dominance analysis further revealed that relationship and conscientiousness could explain 81% variance of PIU, and vitality only accounted for another 19%.

**Conclusions:**

Relationship and conscientiousness were possible protective factors of pathological Internet users, while vitality was vulnerable. The results could be helpful in screening “at-risk” Internet users (low relationship and conscientiousness as well as high vitality). Future intervention strategies could focus on how to enhance relationship and conscientiousness and on how to reduce vitality.

## Background

The International Telecommunications Union (ITU) [1] indicates that the number of Internet users will reach to 2.7 billion by the end of 2013, which accounts for 39% of the world’s population. In the past two decades, the total number of Internet users increased from 0.62 million in 1997 to 564 million by the end of 2012 [2].

Internet use can be likened to a double-edged sword: it provides users benefits and convenience but also brings them harm. For example, a virtual network can provide a safe region for users to relieve social anxiety and shyness [3]; however, it can also cause social isolation and depression [4], especially among adolescents [5]. Among the negative outcomes of inappropriate Internet use, pathological Internet use (PIU) may be the most serious. There have been an increased number of studies on this new mental health problem in the past decades [6]. A recent large-scale study comprised of 11,357 school-based adolescents from 11 counties revealed that the prevalence of PIU was 4.20%, and the PIU was significantly related to suicidal behaviors, depression, attitude problems, and attention deficit hyperactivity disorder [7].

Additionally, the prevalence rates of PIU in adolescents of different countries were considerably high. In European studies, researchers found the rates usually ranged from 1.00% to 9.00% [8,9]; in Asian countries, especially in China, the prevalence rates were even up to 18.00% in adolescents [10-12] and 35.00% in university students [13]. Accordingly, of these Chinese Internet users, around 24.00% or approximately 135.36 million are adolescents [2]; thus, it can be estimated that there are up to 24 million Chinese adolescents are pathological Internet users. This large population and the increasing trend indicated the urgency to investigate the influential factors for effective prevention intervention.

It should be noted, firstly, that there are not any official diagnosis and accepted diagnostic criteria of PIU in recognized diagnostic manuals [14], including the latest DSM-V, and thus different researchers adopted different definitions in their studies. Some researchers defined PIU as the negative impacts of unreasonable or excessive Internet use. It emphasized the cognitive, emotional, behavioural, and physical differences between the regular users and the excessive users [15-18]. Researchers also use Compulsive Computer Use [18], Problematic Internet Use [19], and Internet Dependency [20] to describe the same phenomenon. Although different studies use various terms, they all include the following six core elements: salience, tolerance, withdrawal symptoms, conflict, relapse, and mood alteration [21].

Some models were proposed to explain the causes and mechanism of PIU, such as the problem-behavior theory [22], which focused on the behaviors that trigger the PIU. Within this framework, a study based on 2,114 high school students found that problematic alcohol use was incentive to PIU [23]. However, another study demonstrated that PIU was related to internalizing problems instead of externalizing problems (e.g., substance use and other risky behaviors). The students suffering from social anxiety, depression, and conflicting family relations exhibited a propensity toward PIU [24]. The inconsistency implied that the causes of PIU may due to individual differences [25,26]. Consequently, Davis proposed the cognitive-behavioral model of PIU from the perspective of individual psychopathological etiology [18]. The obvious proximal contributory causes (e.g., stress, danger, cardiac arrhythmia, and substance addiction) and constitutional distal contributory causes (i.e., the diathesis causing psychopathology) were distinguished in this model [27]. Many studies identified several vulnerable personalities among PIUs, such as loneliness [28], low extroversion [29,30], low agreeableness and emotional stability [30], high neuroticism [31,32], and shyness [33,34].

A detailed literature review on PIU will go beyond the scope of the current study; however, based on the above literature, we found that most of the previous studies focused on the negative interpersonal and intrapersonal factors. None of them had a positive perspective. With the flourishing of positive psychology in the recent decade, many psychologists and clinicians became aware of positive factors’ strength on their clients and patients.

Among all of the positive factors, the groundbreaking work was the virtues system proposed by Seligman and his colleagues [35], which included 6 virtues and 24 character strengths [36,37]. Although the subsequent studies have shown that these positive traits are significant to health and well-being in different cultures [38-42], relatively few empirical studies were conducted at the virtue level and the psychopathology area. It may partly due to the issues of virtue assessments and cultural differences [43,44]. For example, based on the 24 character strengths, Kim [45] obtained three-factor virtues using the chronic illness and disability American sample, while Bardar and Kashdan [46] found four-factor virtues with a healthy undergraduate sample. In addition, previous study found that these 24 strengths cannot group into six virtues among Chinese undergraduates [47]. Therefore, Kristjánsson [48,49] suggested that the distinction and selection of the strengths and virtues, as well as the corresponding items, should consider the culturally dependent issues.

In order to solve this issue, Duan et al. [43] adopted the Combined Etic-Emic Approach [50,51] to reduce the culturally inappropriate items of the *Values in Action Inventory of Strengths* (the original questionnaire for measuring the virtues and strengths). Exploratory factor analysis, confirmatory factor analysis, and psychometric evaluation revealed three well-established and culturally meaningful virtues: relationship, vitality, and conscientiousness [52]. Relationship reflects the positive cognitions, emotions, and behavior involved in interactions with others; vitality reflects positive qualities manifested as life forces in the social environment; and conscientiousness reflects intrapersonal traits manifested as psychokinesis in the process of self-regulations [53]. This questionnaire can be used to assess virtues both in Western and Eastern cultures. A preliminary study revealed that vitality was a positive predictor of positive mental health [54].

The present study aims to increase knowledge on virtues by exploring the relationships between virtues and PIU. To our knowledge, this is the first study to examine such a relationship. The results provide insights on virtues and PIU research both in theory and in practice.

## Methods

### Participants and procedures

A convenience sampling method was adopted. Students from 4 junior high schools and 4 senior high schools in 4 provinces (Chongqing, Shanxi, Guangxi, and Jiangsu) of Mainland China were invited to participate in this investigation. Recruitment information was released through the school bulletin board, and all students from year 1 to year 6 (junior high school years 1 to 3 and senior high school years 4 to 6) were invited to participate. Each school was expected to have 100 respondents. The participants were asked to complete a paper-and-pencil questionnaire package and return the completed questionnaires immediately.

Several methods were adopted to prevent the common method bias [55]. Four questionnaire packages were prepared (A, B, C, and D). The order of appearance of items in each package was different. Participants were randomly assigned a package for completion. Next, participants were asked to complete the package during their free time. Data was collected in different areas of Mainland China, including eastern, central, and western regions. Informed consent was obtained prior to participation. Ethics approval of the project was obtained from the Department of Psychology and Culture Studies, Research Institute of Chongqing Culture Development, Chongqing, China. Before we started to collect data, the research proposal and ethics approval were sent to be reviewed by Mental Health teachers in the target schools. The teachers are psychological professionals in Chinese campuses and are responsible for students' psychological health education. Informed written consent from the teachers was obtained. Ethics committees also approved this consent procedure. All the process of data collection was overseen by the mental health teachers to guarantee the rationality, legitimacy, and effectiveness of data collection.

Data were collected from February 2013 to July 2013. A total of 674 valid participants voluntarily attended and completed this investigation, and the response rate was 84.25%.

### Measurements

#### Adolescent Pathological Internet Use Scale (APIUS)

APIUS is a 38-item simplified Chinese scale for measuring PIU among adolescents [56]. It was developed based on Young’s [16,21,28] and Davis’ [18] theories and includes six dimensions: salience, tolerance, compulsive Internet use/withdrawal symptoms, mood alteration, social comfort, and negative outcomes. The participants were asked to rate the consistency on each item (from “1 = totally inconsistent” to “5 = totally consistent”). The severity of PIU is represented by the mean score of the entire scale, and a high score reflects high severity. The Cronbach’s reliability *α* (.95), four-weeks test-retest reliability (.86), and criterion validity (.38 to .77) were satisfactory according to the original developer [56]. Based on the previous studies, the scale developer [56] also suggested that a mean score of 3.15 be adopted as a cut-off to screen for Internet addiction. In the current study, the Cronbach’s *α* of the entire scale was .91. Although there were 21 other self-report instruments to screen PIU [57], such as the Internet Addiction Test, Young’s Diagnostic Questionnaire, and the Chen Internet Addiction Scale, Beard [17] found that these measurements had limited consensus on the underlying dimensions [14]. Therefore, in such a case, an etic-based instrument was a better choice for considering the characteristics of targets.

#### Chinese Virtues Questionnaire (CVQ)

CVQ [43,52] is a simplified Chinese scale that assesses three virtues: relationship (32 items), vitality (40 items), and conscientiousness (24 items). The respondents were asked to rate the extent to which each item described them on a five-point Likert scale ranging from 1 (very much unlike me) to 5 (very much like me). The mean scores of the three virtues were obtained by summing the corresponding items of each subscale and then dividing them by the number of items. A high score reflects a high degree of the virtue within an individual. The coefficients of internal reliability of the total questionnaire (*α =* .95), relationship subscale (*α =* .92), vitality subscale (*α =* .93), and conscientiousness subscale (*α =* .89) in the present sample were a positive find.

### Data analysis

The virtues and PIU were calculated by mean scores. Statistical analyses were performed on SPSS 20.0. T-tests were used to compare the virtues of the male sample and female sample, as well as the PIU sample and the non-PIU sample. It was also used to reveal the difference of virtues between the PIU sample and the non-PIU sample. Pearson correlation analyses were performed to obtain the correlation coefficients among these psychological variables. Multivariate regressions were performed to assess the association between different virtues and PIU. Thereafter, dominance analysis was conducted to identify the relative contribution of each virtue when PIU was predicted. A significant difference was considered to exist: p < 0.01.

## Results

### Demographic results

A total of 302 (44.80%) participants were males and 372 (55.20%) participants were females. The mean age was 15.10 years (*SD* = 1.81, ranging from 12 to 17). A total of 296 (43.92%) students were from junior high school, while 378 (56.08%) came from senior high school. The average length of Internet use was 5.31 years (*SD* = 2.09). It should be noted that such a sample size might be small for epidemiological investigations; however, the focus of the current study was to examine the relationship among psychological variables. Accordingly, this sample size was appropriate and acceptable for this study [58].

### Descriptive statistics and difference analysis

According to the cut-off point of APIUS (re *Measurements section*), 64 students (9.50%) had PIU mean scores above 3.15 and were defined as the PIU group, while the remaining students were defined as the non-PIU group. The descriptive statistics of all variables of the different groups were shown in Table 1. Among the three virtues in different subgroups, relationship had the highest scores (*mean* = 3.89 to 3.97), followed by vitality (*mean* = 3.53 to 3.55) and conscientiousness (*mean* = 3.11 to 3.29). Relationship in females was significantly higher than in males (*t* = 2.60, *p* = 0.01); conscientiousness in the PIU group was significantly lower than that in the non-PIU group (*t* = -2.18, *p* < 0.01). The PIU scores were significantly different between males and females (*t* = 3.86, *p* < 0.01).

**Table 1:** Descriptive statistics and difference analysis of variables for different sub-groups

		**Gender**			**Diagnosis**		
	**Total sample**	**Male**	**Female**	***t***	**PIU**	**Non-PIU**	***t***
	***M (SD)***	***M (SD)***	***M (SD)***		***M (SD)***	***M (SD)***	
Relationship	3.91(.42)	3.85(.42)	3.97(.41)	-2.60*	3.89(.48)	3.92(.42)	-.30
Vitality	3.54(.48)	3.55(.48)	3.53(.48)	.34	3.55(.53)	3.54(.48)	.17
Conscientiousness	3.27(.44)	3.28(.40)	3.27(.47)	.24	3.11(.52)	3.29(.43)	-2.18*
PIU	2.36(.59)	2.50(.62)	2.25(.55)	3.86**	-	-	-

*p < .01; **p < .001.

### Correlations and regression analysis

Table 2 shows the Pearson correlation coefficients. The PIU scores were negatively related to relationship (*r* = -.19) and conscientiousness (*r* = -.19). Regression analysis was conducted to explore the role of virtues in affecting PIU. The PIU was set as a dependent variable, and three virtues (relationship, vitality, and conscientiousness) were set as independent variables. Relationship (*β* = -.24) and conscientiousness (*β* = -.21) can negatively predict PIU, while vitality (*β* = .25) can positively predict PIU.

**Table 2:** Correlations analysis for all variables

	**Relationship**	**Vitality**	**Conscientiousness**
Salience	-.11*	-.02	-.17**
Tolerance	-.20**	-.04	-.15**
Compulsive internet use	-.18**	-.07	-.17**
Mood alteration	-.06	.04	-.09
Social comfort	-.05	.04	-.05
Negative outcomes	-.28**	-.20**	-.23**
PIU	-.19**	-.05	-.19**

*p < .01; **p < .001.

### Dominance analysis

Dominance analysis was conducted to reveal the relative importance of three virtues for PIU. Usually, researchers used multiple regression analysis to calculate the regression coefficients for each independent variable and then determined their relative importance by comparing these regression coefficients. However, JW Johnson [59] suggested that the traditional multiple regression analysis, including stepwise regression and hierarchical regression, may overestimate the predictive power of the stronger independent variable or may underestimate the predictive power of the weaker independent variable. According to this perspective, DV Budescu [60] proposed the dominance analysis to refine the current approaches.

Three virtues in the current study may construct 7 different combinations, i.e., three single-variable combinations, three two-variable combinations, and one three-variable combination. The PIU was set as a dependent variable, and 7 combinations entered the regression equations. A total of 7 regressions were conducted (re Table 3). The relative contribution of each variable was recalculated based on the obtained *R*^*2*^ values (re the penultimate line, Table 3). Finally, the three virtues’ relative contribution (*R*^*2*^) was divided by .073 to assess the relative importance of each predictor. In the current study, we found that relationship contributed 42.47% of the predicted variance, following by conscientiousness (39.73%) and vitality (19.18%).

**Table 3:** Dominance analysis

	***R***^***2***^	**Relationship**	**Vitality**	**Conscientiousness**
	-	.036	.002	.036
Relationship	.036	-	.012	.010
Vitality	.002	.046	-	.043
Conscientiousness	.036	.010	.009	-
Relationship and Vitality	.048	-	-	.025
Relationship and Conscientiousness	.046	-	.027	-
Vitality and Conscientiousness	.045	.028	-	-
Relationship, Vitality, and Conscientiousness	.073	-	-	-
Decomposition of *R*^*2*^		.031	.014	.029
% of the Predicted Variance		42.466%	19.178%	39.726%

## Discussion

The aim of the present study is to explore the relationship between virtues and PIU for an in-depth understanding of virtue theory and Internet addiction. The results suggested the possible protective role of relationship and conscientiousness, which contributed 82.20% of the predicted variances. Although vitality was a vulnerability to PIU, it was less important (19.18%) compared to the other virtues.

Previous studies had identified the stable and strong correlations between personal relationships and PIU. However, these studies often took relationships as outcome variables. For example, Milani et al. [61] found that adolescents who used the Internet many hours a week had dysfunctional coping strategies and poor interpersonal relations. The results of the present study, which are consistent with previous studies from different perspectives, reveal that the individuals with strong relationship may have fewer PIU symptoms. That means a person with strong relationship often leads to good interpersonal relations, and it further prevents the individual from seeking substitutes in a virtual network [62]. The second meaningful protective trait was conscientiousness, which negatively predicted PIU in the current study. Karim, Zamzuri, and Nor [63] reported that conscientiousness in Big Five model was significantly and negatively correlated with unethical Internet behavior in university students. Another meta-analysis indicated that conscientiousness-related traits were negatively related to risky health-related behaviors [64]. Conscientiousness reflects the Chinese traditional cultural concept of “*shendu*” (慎獨), which emphasizes that no matter what the circumstance is, individuals should impose self-restriction and control their own behavior. According to this idea, individuals with high conscientiousness should control their emotions, cognitions, and behaviors and display fewer incidents of improper or excessive use of the Internet, thereby inhibiting the emergence of Internet addiction.

A recent study conducted by Akın [65] demonstrated that subjective vitality negatively predicted Internet addiction; further analysis revealed that subjective vitality can mediate the relationship between Internet addiction and subjective happiness. Other previous studies also revealed the positive and protective role of vitality [43,52,54]. However, the present results found the opposite effect of vitality, which recognizes vitality as a vulnerability to PIU. Ko et al. [66] thought that the novelty seeking or sensation seeking [67] of an individual was one of the reasons that caused Internet addiction. Our vitality virtue included other sub-traits such as curiosity, bravery, and creativity. So far, this result has rarely been repeatedly reported. More studies in the future are needed to explore the difference between these different “vitality” concepts. Suler [68] also believed that healthy or pathological Internet use was determined by the fulfillment of basic psychological needs, while adolescence was a special period in developmental psychology. During this period, individuals are full of vitality and exhibit a strong desire, need, and interest to explore the unknown world [69]. Therefore, it will be beneficial and interesting to conduct such longitudinal studies.

Another issue, not directly related to our result but related to the topic, should be noted. The fifth edition of the Diagnostic and Statistical Manual for Mental Disorders (DSM-5) has already identified “Internet Gaming Disorder” in Section III (Emerging Measures and Models). It suggested that more clinical researches and experience should be obtained before the “Internet Gaming Disorder” could be recognized as a formal disorder. However, it should be noted that the PIU used in the current study is a very broad concept, which concerns a series of unhealthy outcomes caused by excessive Internet use, including general use of the Internet, social media, gambling, porn, as well as games. Therefore, more studies should be performed to examine whether the virtues play the same role in different Internet activities.

Some other limitations should be noted. First, the sample size is relatively small compared to an epidemiology study. Hence, the prevalence of PIU in the current sample should not be generalized. In addition, the reported prevalence rates differ may be regarded as a consequence of different assessment tools and cut-offs [57]. Second, only self-reporting measurements were adopted in the current study. An increasing amount of non-responder indicators should be adopted in future pathological Internet use studies to reveal a more objective profile. Finally, this is only a preliminary investigation. Future studies should take other factors, such as social support, curiosity and self-regulation, into account to further clarify the mechanism behind the relationship between virtues and PIU.

## Conclusion

The relationship and conscientiousness are possible protective factors for PIU adolescents, while vitality may be the risk factor. The current results will facilitate the development of treatments and intervention for Internet addictive patients and will serve as pre-intervention for risk groups (high vitality adolescents). After all, there is a need to transport the scientific evidence established in the studies into actual clinical practice.

## Competing interests

The authors declare that they have no competing interests.

## Authors’ contributions

YZ, ZY and WD conducted and coordinated the study. WD, ZY and YZ performed the statistical analysis and drafted the manuscript. YZ and XT contributed critical comments on the manuscript. FGan, FW, JW, YW, and FGuo collected the data with the other authors. All authors have read and approved the final manuscript.

## Authors’ information

Yonghong Zhang and Zhihan Yang are the co-first authors of this paper.
